# Controversial Impact of Sirtuins in Chronic Non-Transmissible Diseases and Rehabilitation Medicine

**DOI:** 10.3390/ijms19103080

**Published:** 2018-10-09

**Authors:** Alessia Mongelli, Carlo Gaetano

**Affiliations:** ICS Maugeri S.p.A., SB, via Maugeri 10, 27100 Pavia, Italy

**Keywords:** epigenetics, rehabilitation, DNA methylation, histone modification, HDAC, exercise, health span, heart failure, neurodegeneration, cancer, lung fibrosis, bone formation

## Abstract

A large body of evidence reports about the positive effects of physical activity in pathophysiological conditions associated with aging. Physical exercise, alone or in combination with other medical therapies, unquestionably causes reduction of symptoms in chronic non-transmissible diseases often leading to significant amelioration or complete healing. The molecular basis of this exciting outcome—however, remain largely obscure. Epigenetics, exploring at the interface between environmental signals and the remodeling of chromatin structure, promises to shed light on this intriguing matter possibly contributing to the identification of novel therapeutic targets. In this review, we shall focalize on the role of sirtuins (Sirts) a class III histone deacetylases (HDACs), which function has been frequently associated, often with a controversial role, to the pathogenesis of aging-associated pathophysiological conditions, including cancer, cardiovascular, muscular, neurodegenerative, bones and respiratory diseases. Numerous studies, in fact, demonstrate that Sirt-dependent pathways are activated upon physical and cognitive exercises linking mitochondrial function, DNA structure remodeling and gene expression regulation to designed medical therapies leading to tangible beneficial outcomes. However, in similar conditions, other studies assign to sirtuins a negative pathophysiological role. In spite of this controversial effect, it is doubtless that studying sirtuins in chronic diseases might lead to an unprecedented improvement of life quality in the elderly.

## 1. Background

Sirtuins (Sirts) belong to the III class of histone deacetylases. They are evolutionally conserved and require NAD^+^ as cofactor. Sirts deacetylate lysines in histone and non-histone proteins and generate a molecule of nicotinamide and one of *O*-acetyl-ADP ribose. Importantly, in response to stress conditions Sirts’ function as cellular redox sensor and activate protection mechanisms. They can be nuclear, cytosolic or mitochondrial. Sirt1 is probably the best characterized among all sirtuins however its role in pathophysiological conditions is still unclear. Specifically, in cancer cells, the role of Sirt1 is controversial. Some evidences suggest a tumor suppressor activity, while other observations confer to them an oncogenic potential. This heterogeneous response seems context-dependent. Noteworthy, in some tumors, such as breast cancer, an intense physical exercise decreases tumor biomarkers. In cardiovascular disease (CVD) cardiac rehabilitation often combined to a pharmacological treatment achieves significant positive effects. At molecular level, physical training induces an increase in Sirt1 and antioxidant catalases while superoxide dismutase decreases resulting in reduction of intracellular reactive oxidant species (ROS) production. In other conditions, such as chronic obstructive pulmonary disease (COPD), physical exercise ameliorates clinical symptoms and increases Sirt1 activity that negatively regulate NFκB pathway and metallo-proteinases secretion significantly decreasing inflammation. In neurological disorders such as Alzheimer disease (AD), Sirt1 is decreased. As a consequence, Tau protein is acetylated and accumulates. The physical exercise enhances Sirt1 activity reducing Tau storage and Sirt3 has a neuroprotective effect deacetylating mitochondrial p53. In Parkinson’s disease (PD) Sirt1 level is down-regulated by Cdk5 through ubiquitin-proteasome pathway that is up-regulated. After physical exercise Sirt1 and mitochondrial complex I activities increase, and PD symptoms are ameliorated. In Huntington disease (HD), Sirt1 is not able to deacetylate TORC1 that in consequence does not bind CREB, due to the presence of mutated huntingtin protein interfering with TORC1-CREB complex. As a result, factors that regulates neuronal growth are not transcribed. Physical rehabilitation program has been reported to increase Sirt1 expression improving HD symptoms. However, this is a palliative intervention, waiting for the definitive genetic correction of the disease.

## 2. About Sirtuins and Their Pleiotropic Properties: An Overview

Sirtuins (Sirts) are a family of evolutionarily conserved class III histone deacetylases originally discovered in *S. Cerevisiae*, named Silent Information Regulator 2 (Sir2) and conserved to humans [1]. Biochemically, they act predominantly as ADP-ribosyl-transferases requiring NAD^+^ and an acetylated lysine substrate to produce a molecule of nicotinamide and *O*-acetyl-ADP ribose, which in turn can be used as a metabolic messenger by the cell [2,3]. The mammalian Sir2 family is composed by seven members (Sirt1-7) that differ from each other for specificity, catalytic activity and cellular localization depending on cell, tissue type, metabolism and stress conditions. All Sirts share a conserved domain called Sirtuin Core Domain, formed by 250–260 amino acids, which retain catalytic activity and NAD^+^-binding site. Sirts differ at their N and C terminus domains that confer differences in molecular weight, and perhaps substrate specificity. These HDACs are involved in multiple functions controlling chromatin structure and integrity [4], inhibition of senescence and apoptosis [5], metabolic homeostasis [6], cell differentiation and development. Despite all of these activities, Sirts’ main role seems that of cellular redox sensors. In response to stress signals, in fact, they get activated and most of them deacetylate lysine residues on histones, especially H4K16Ac and H3K9Ac [7], but they can also remove acetyl groups on non-histone proteins, such as p53 [8] and tubulin [9]. More in detail, Sirt1, the best characterized Sirt member, is the only one with a clear role in genomic silencing through its effect on heterochromatin formation. The Sirt1 main biochemical form is that of a homo-trimer, which is localized predominantly in the nucleus [10]. Here, Sirt1 deacetylases not only H4K16Ac and H3K9Ac, but also H1K26Ac [10], the di-trimethyltransferase Suv39h1 [11], FOXO [12], NFκB [13], p53 [14], Ku70 [15], eNOS [16], PGC-1α [17], MyoD, PCAF [18], UCP2 [19] and PPARγ [20] to name few of the most common targets. As a result, cell survival and DNA repair are enhanced. In addition, in some pathophysiological conditions, including cardiac hypertrophy and tumor progression, Sirt1 clearly activates protective signaling pathways controlled by AKT and PDK1 [21].

The main function assigned to another Sirt member, Sirt2, at least in HeLa and HEK293T cells, is the deacetylation of cytosolic α-tubulin via HDAC6 interaction [9], which causes inhibition of the active intracellular transport [22]. Besides this cytosolic function, a temporary nuclear Sirt2 localization was found during G2/M transition marked by H4K16Ac deacetylation [7]. Moreover, in mouse erythrocytes, it has been demonstrated that Sirt2-dependent deacetylation activates glucose six phosphate dehydrogenase (G6PDH), which stimulates the pentose phosphate pathway to supply cytosolic NADPH under oxidative stress [23].

In response to the insulin signal, Sirt2 is activated by AMPK stimulating Akt and leading to its deacetylation and activation [24]. Noteworthy, Sirt2 has been found involved in the regulation of some other biological stress-tolerance pathways. When Sirt2 is down-regulated, in H9c2 cells, the chaperonin 14-3-3 ζ is increased and sequesters the pro-apoptotic protein BAD into the cytoplasm preventing localization to mitochondria, a necessary step towards apoptosis [25]. In vivo, Sirt2 inactivation has been found to deacetylate and stabilize BubR1, a mitotic checkpoint kinase, which activation leads to an increase in lifespan [26]. 

Mitochondria are another relevant cellular compartment, in which Sirts play important regulatory roles. Sirt3 deacetylates some mitochondrial proteins involved in crucial metabolic pathways [27]. For example, under stress conditions, Sirt3 migrates from the nucleus to mitochondria where, by deacetylation, this sirtuin increases the enzymatic activity of Acetyl-CoA Synthase 2, which becomes active and enhances the ratio of mitochondrial/nuclear protein localization in favor of mitochondria [27]. In normal growth conditions, Sirt3 coexists in both nuclear and mitochondria compartments [28], but in the nucleus, Sirt3 is predominantly associated with Sirt2 contributing to H4K16Ac deacetylation and chromatin remodeling [28].

Sirt4 and Sirt5 are other mitochondrial specific sirtuins. In particular, Sirt5 acts as a protein modifier removing succinyl and malonyl groups from lysine [29,30]. Similarly, Sirt4 does not have any deacetylating function, but it acts on mitochondrial proteins, such as glutamate dehydrogenase (GDH), as ADP-ribosylase. In consequence of Sirt4 function, GDH results inactivated [31]. 

Sirt6 and Sirt7 are localized into the nucleus where their activity is that of an ADP-ribosyltransferase. Specifically, Sirt7 is nucleolar and interacts with RNA Polymerase I for the transcription of ribosomal genes [32], activates the nuclear respiratory factor (NRF) isoform α/β complex via deacetylation of NRFβ1 regulating mitochondrial biogenesis and function [33]. Sirt7 also activates the testicular nuclear receptor 4 (TR4), which transcribes genes involved in lipid metabolism increasing fatty acid uptake and triglyceride synthesis/storage [34]. Meanwhile, Sirt6 is involved in genomic integrity, it is a chromatin-associated deacetylase and is ubiquitously expressed from early to adult stages [35]. Sirt6 interacts with a putative tumor suppressor and negative regulator of cell proliferation known as cyclin D1 binding protein 1 GCIP factor. As a result, Sirt6 stimulates DNA repair or proliferation arrest when genomic integrity is compromised [36]. 

## 3. Sirtuins and Physical Exercise in Cancer

The role of Sirt1 in cancer remains unclear probably in consequence of the heterogeneity that characterizes the disease. Some evidences, in fact, support a Sirt1 role as tumor suppressor while others suggest for an oncogenic potential. In colon cancer, Sirt1 suppresses cancer development via a feedback loop, in which c-Myc is involved. c-Myc binds Sirt1 gene promoter increasing sirtuin expression, while Sirt1 deacetylases c-Myc that loses its DNA affinity [37]. This tumor suppressor activity has been confirmed in vivo after Sirt1 overexpression in a mouse model of colon cancer. In this setting, the animals showed a four-fold reduction in size and number of adenomas in the small intestine and colon [38]. On the contrary, in human colon cancer Sirt1 is often overexpressed, especially in advanced stages [39]. This contradiction might be perhaps explained by Sirt1 deacetylase activity on p53, which determines a weak DNA-p53 interaction that might cause loss of DNA repair capacity [14]. Furthermore, Sirt1 negatively controls the expression of p16/INK4A while promoting Rb phosphorylation, cell proliferation and reduction in cellular senescence as seen in human embryonic lung fibroblasts [40]. Recently, it has been demonstrated that Sirt1 has a pathogenic role in breast cancer, in which it is upregulated promoting cell proliferation. In this context, Sirt1 modulates the p-AKT/AKT ratio in favour of the phosphorylated form of the molecule, which becomes active and promotes cellular division. Again, in breast cancer Sirt7 expression has been found elevated in early disease stages compared to advanced cancer [41]. In addition, prostate, bladder carcinoma, glioblastoma, and ovarian cancers with mutated BRCA1, all show a reduction of Sirt1 expression compared to normal tissues [42]. The biological consequences of this down-regulation are still unclear. Interestingly, it has been reported that physical activity has a positive effect on people affected by colon cancer. A study reports that the beneficial effects of exercise are dose-dependent [43]. Aerobic exercise up to 300 min per week, in fact, significantly decreased tumor biomarkers, such as the prognostic vascular adhesion molecule 1 compared to patients whose followed a low dose exercise program [43]. Whether this positive effect on cancer patients might be dependent on sirtuins activation it is still controversial. However, the activation of sirtuins that occurs upon exercise might play an important role in this condition. In fact, in spite of its apparent pro-oncogenic properties, Sirt1 seems important for an efficient DNA-break repair. Cells and mice knocked down for Sirt1, exhibited DNA damage–induced aneuploidy with an efficiency of DNA break repair reduced by approximately 50% [42]. Based on these evidences, although still very speculative, the genetic targeting by siRNA or other approaches of Sirt1, Sirt7 or other Sirts in combination with physical exercise might represent an innovative therapeutic approach potentially beneficial in cancer (see Table 1). This is an important question worth of further investigations. In line with this consideration, in a recent meta-analysis, Sirt3 emerged as a negative prognostic biomarker for tumors, such as chronic lymphocytic leukemia, breast cancer, colon cancer, hepatocellular carcinoma and renal carcinoma [44]. For Sirt4, 5, 6, and 7, there is only incomplete evidence about whether they may be relevant in cancer or not [45].

## 4. Exercise and Sirtuins in Cardiovascular Diseases

Of the 56.4 million estimated worldwide deaths in 2015, about 54% occurred in consequence of cardiovascular diseases (CVD) [46]. In this context, it has been clearly shown that physical activity and a healthy lifestyle significantly decrease the risk of CVDs and their worsening [47]. Accordingly, a large number of cardiac rehabilitation (CR) programs have been developed to treat CVDs in an attempt of preventing secondary events. In CVDs, CR consists of at least three phases [48]. The first, is performed during the acute phase, and is aimed at preventing/reducing immediate complications. In a second phase, operators stabilize patient’s symptoms and conditions. Finally, patients enter in a long term CR and follow-up program [48]. Nowadays, the most important CVD that may interest old people is chronic heart failure (CHF) and a body of literature demonstrates the beneficial effects of physical activity in this category of patients [49]. In fact, to take under control this severe pathophysiological condition, for which an effective cure does not exist, it is important to follow a detailed exercise program at least for two/five times per week, 45/60 min each session [49]. After this training, an improvement of the quality of life and physical function has been consistently reported [49]. Notably, in untrained CHF patients, Sirt1 in significantly down-regulated, a phenomenon that has been seen also in cardiac hypertrophy and in the presence of pressure overload [50]. In a recent study, the beneficial effect of exercise in CHF patients has been investigated throughout and at the end of an intense rehabilitation program. Blood samples were collected to evaluate Sirt1, superoxide dismutase (SOD) and antioxidant catalase (Cat) activity in peripheral blood mono-nucleated cells and in plasma samples respectively [51]. The result of this trial showed an increase of Sirt1 and Cat compared to control levels, while SOD was reduced [51]. Additionally, this study evaluated the effect of patients’ serum on the senescence process of endothelial cells (EC). Specifically, ECs were challenged with serum of HF patients taken at the end of rehabilitation training. In this condition, a strong reduction of senescence biomarkers was observed compared to controls [51]. To better understand the role of Sirt1 in this process, Sirt1 was inhibited determining an increase of EC senescence and a decrease in Cat activity [51]. These data clearly contribute the evidence that Sirt1 is activated during CR in CHF patients, however, further experiments on a larger cohort of subjects should be performed to better understand the contribution of Sirt1 in CHF.

Biologically, Sirt1 results pivotal during heart development, especially during the second heart field formation, in which Sirt1 silences ISL1 driving the differentiation of cardiac progenitor cells into cardiomyocytes [52]. Afterwards, Sirt1 level declines by at least 80% since the beginning of organogenesis, remaining at that level during adulthood [53]. In contrast, Sirt1 remains highly expressed in endothelial cells, in which it upregulates endothelial nitric oxide synthase (eNOS) expression and function, contrasting the development of atherosclerosis, smooth muscle cells senescence, inflammation and accumulation of ROS in arteries thus supporting vascular growth [16]. Once activated, Sirt1 reduces cholesterol biosynthesis in hepatocytes and macrophages [54] and upregulates the X receptor (LXR) in liver [55]. The deacetylation mediated by Sirt1 on LXR results in a decrease of plasma HDL levels, due to enhancement of reverse cholesterol transport and balancing of fatty acids metabolism, which lead to a global reduction lipids in serum [55]. Moreover, in macrophages and endothelial cells a functioning Sirt1 decreases expression of inflammatory molecules, including TNFα, intercellular adhesion molecule (ICAM)-1, interleukin (IL)-6, IL-1, and inducible NOS (iNOS) [56,57]. In this context, when Sirt1 deacetylases FOXO (a negative regulator of blood vessels) this transcription factor became inactive and the generation of new blood vessels is facilitated [58]. Other evidence shows that Sirt1 is downregulated during ischemia/reperfusion injury (I/R) [59]. In experiments performed on cardiac specific Sirt1-deficient mice, the animals exhibited heart damage similar to that caused in humans by I/R [60].

In spite of the unquestionable evidence about a positive role for Sirt1 activation during heart development or in the slowdown of CVDs worsening during adult life, Sirt1 upregulation has also been associated with the onset of pathophysiological conditions, such as those determined by nutrient starvation, pressure overload, and ischemic preconditioning. In pressure overload, nuclear Sirt1 activates PPARα, which binds to the estrogen-related response element, resulting in the repression of genes controlling mitochondrial function and cardiac contraction [50]. In cardiac hypertrophy, Sirt1 may act in the cytosol, where it deacetylases Akt and PDK1, PDK1 phosphorylates Akt, which becomes active and promotes hypertrophy [21]. Consistently with these observations, in Sirt1-deficient mice it has been shown that after physical exercise cardiac hypertrophy was prevented or reduced [21]. Furthermore, in the presence of heart failure it has been demonstrated that the Sirt1-PPARα complex is able to downregulate targets of the estrogen related receptor response element targets promoting mitochondrial dysfunction [50]. Hence, similarly to what has been observed in cancer, Sirt1 might have protective or deleterious effects on the heart depending on the specific pathophysiological environment or in consequence of specific stressor triggers, such as lipids. To achieve positive therapeutic effects, additional experience seems necessary in order to understand under what condition Sirts function is beneficial and when, instead, they should be silenced.

To pharmacologically modulate Sirts activity, some derivatives of natural molecules are other synthetic products are now available. At present, only natural products are approved for human use. Resveratrol (Res) is so far the best characterized Sirts activator. It is a polyphenol found abundant in grapes, blueberries, raspberries, and mulberries, which seems to have a protective effect on the heart [61]. Whether this protective effect depends on the activation of Sirts remains unclear. However, it is clear that through direct and indirect mechanisms, Res may stimulate Sirt1 to exert PGC-1α deacetylation [62] achieving improved fatty acid storage and glucose metabolism regulation. As part of its effects, Res can activate AMPK, which directly phosphorylates eNOS causing an increase of nitric oxide (NO) production, vessel dilatation [63] and reduction of ROS levels. In fact, a Res-activated Sirt1 physically interacts with eNOS and deacetylates lysines in the calmodulin-binding domain of eNOS, leading to enhanced eNOS activity [64]. Recently, it has been demonstrated the Res activity on renin-angiotensin system, in which the polyphenol decreases the activity of prorenin receptor (PRR)-angiotensin converting enzyme (ACE)-Ang II axis in the aorta. The action of Res, enhancing ACE2-Ang-(1 and 7)-Ang II type 2 receptor (AT2R)-Mas receptor (MasR) axis, determines a cardiovascular positive effect [65]. The combination of Res and exercise has been found beneficial on vascular function reducing inflammatory markers and enhancing vessel responsiveness [66]. However, a recent work reported that the beneficial effect of resveratrol could be due to an antioxidant effect without any evidence of Sirt1 activation [67].

For reasons still undetermined, Sirt2 has been found important maintaining cardiac homeostasis while counteracting hypertrophy and failure. In fact, during pathological hypertrophy, Sirt2 levels were significantly reduced in the human heart, and this observation has been confirmed by Sirt2 deficient animals, in which agonist-induced hypertrophy was exacerbated. In this condition, Sirt2 overexpression significantly attenuated hypertrophy in cardiomyocytes [68]. In case of Sirt2 reduction, cardiac remodeling apparently occurred in consequence of missing interaction between Sirt2, that cannot migrate into the nucleus in G2/M phase, and the transcription factor NFAT isoform C2 (NFATc2), which is not deacetylated resulting in increased transcription activity of foetal cardiac genes contributing to hypertrophy [68]. In vitro, the downregulation of Sirt2 in embryonic myoblasts enhanced anoxia-reoxygenation stress tolerance sequestering Bad and contrasting the progression of cell death [25]. In HeLa and HEK-293T cells, it has been reported that Akt binds directly Sirt2 instead of PI3K activating Akt pathway during glucose deprivation [24]. Moreover, the effect of Sirt2 on Akt activation, similar to that of Sirt1, suggests that its activation could prevent cardiac hypertrophy [24]. However, as for other aforementioned pathophysiological conditions, in adult tissues the protective role of Sirt2 is not well characterized yet.

In consequence of its important mitochondrial function, Sirt3 has been linked to congenital heart diseases characterized by mitochondrial dysfunction. It has been shown, in fact, that in Friedreich’s ataxia (FRDA), an early onset (at 10–15 years of age) autosomal recessive disease characterized by low levels of NAD^+^, there is not enough Sirt3 activity. The normalization of the NAD^+^/NADH ratio in FRDA increases Sirt3 function contributing to the amelioration of cardiac function. This evidence suggests that Sirt3 can be a therapeutic target in pediatric FRDA patients [69]. In this direction, the mouse model of Sirt3 deficiency shows that the increase of mitochondrial transition pore permeability (mPTP) [70] paralleled by a dysregulated TGFβ1 signaling [71] is associated with cardiac hypertrophy and interstitial fibrosis detectable since early life stages. 

In the presence of heart hypertrophy, Sirt3 results overexpressed causing an increase of MAPK/ERK, PI3K/Akt and Ras pathway activity, as well as an intracellular increase in ROS levels. In consequence, genes involved in cell growth are activated while antioxidant genes, such as manganese superoxide dismutase (MnSOD) and Cat, become repressed [72]. In contrast, a protective effect of Sirt3 on cardiomyocytes apoptosis has been observed upon overexpression. Sirt3, in fact, may act on Ku70, which sequesters the pro-apoptotic protein Bax out from mitochondria [73]. Furthermore, Sirt3 contrasts doxorubicin-induced cardiomyopathy activating the *O*-guanine-DNA glycosylase-1 (OGG1), an important mitochondrial DNA repair enzyme [74]. Again, it appears that the protective or detrimental effects of Sirt3, as for other members of the family, might depend on the specific pathophysiological context.

Little is known about Sirt4 and Sirt5 in CVDs. However, their protective effects on neonatal cardiomyocytes have been recently reported. Specifically, Sirt4 overexpression in H9c2 cells leads to an increase in procaspase/caspase ratio and a decrease of Bax translocation to mitochondrial membrane [75]. Sirt5 knockdown, instead, decreases the viability of neonatal cardiomyocytes, enhancing the activity of caspase 3 and 7. In the presence of oxidative stress, Sirt5 binds Bcl-xl blocking the apoptotic cascade and increasing cell survival [76] thus suggesting the regulation of its expression as a novel treatment of the oxidative stress-related cardiac injury. 

Sirt6 has been found to be downregulated in failing human hearts. Sirt6 is a key regulator of cardiac hypertrophy and heart failure controlled by IGF-Akt signaling through c-Jun and the deacetylation of histone 3 (H3) at lysine (K) 9 [77]. It has been shown, in fact, that Sirt6 overexpression protects against cardiac hypertrophy by physical interaction with c-Jun, which becomes inactivated and the transcription of genes involved in IGF signaling is inhibited [77]. On the other hand, in a condition of Sirt6 deficiency, the hyper-acetylation of H3K9 increases c-Jun activity and cardiac hypertrophy worsening heart failure [77]. In hypoxia, the overexpression of Sirt6 activates AMPK pathway, increases Bcl2 protein level, inhibits NFκB, decreases ROS formation and p-Akt activity [78]. In this condition, cardiomyocytes are protected from hypoxic stress and apoptosis [78]. Moreover, in the presence of oxidative stress, Sirt6 deacetylates and ADP-ribosylates PARP1 activating non-homologous end joining and homologous recombination repairing of DNA double-strand breaks (DSB) [79]. 

About Sirt7, it has been associated with a cardioprotective role in consequence of its effects on Akt and p53 in cardiomyocytes [80]. In mice, it has been shown that reduction of Sirt7 expression and activity leads to Akt and p53 hyper-acetylation causing cardiac hypertrophy and cardiomyopathy accompanied by extensive fibrosis associated to heart failure [80]. However, the overall picture is still too fragmented, and little or no information is available about the effect of physical exercise on sirtuins in general and specifically on Sirt4, 5, 6, and 7 (see Table 2). 

## 5. The Involvement of Sirtuins in Other Pathophysiological Conditions Relevant to Rehabilitation Medicine: The Chronic Obstructive Pulmonary Disease (COPD)

The chronic obstructive pulmonary disease (COPD) is a progressive lung disease that includes emphysema, chronic bronchitis, refractory (non-reversible) asthma, and some forms of bronchiectasis. COPD is characterized by progressive and largely irreversible airflow limitation, which is associated in the lung to an abnormal inflammatory response. Cigarette smoke (CS) is the major risk factor for COPD. CS, in fact, increases the inflammatory state in lungs even in smokers without COPD [81]. Among other risk factors, a major role is assigned to the environment (dust, air pollution, and second-hand smoke) or to associated genetic conditions, such as the α-1 deficiency-related emphysema [82]. At present, COPD treatment is aimed at controlling/reducing dyspnea by using bronchodilators [83]. Further, these patients can be admitted to a pulmonary rehabilitation program, in which they follow a specific physical activity made of postural and aerobic exercises, breathing and respiratory muscle training and upper- and lower-body muscle strength exercises [84]. At the end of such a training program some parameters are measured to compare with the original values [84]. Usually, dyspnea, duration of the exercise, walking distance and pulmonary capacity are all objectively improved and all patients report about a general amelioration of their clinical symptoms [84]. Of interest for this review, a recent study reported about a correlation between the physical effect determined by COPD rehabilitation programs and the presence of specific epigenetic modifications [85]. After an intense program of training sessions, in fact, it was found that HDACs activity increased [85], as well as increased all anti-inflammatory signals associated with TGFβ reduction and upregulation of IL-6 [85].

A progressive worsening of inflammation is typical in COPD patients. Sirt1 seems to play a role in this context negatively regulating NFκB through its deacetylation and inactivation. Sirt1 expression, in fact, decreases under the effect of cigarette smoke, facilitating the release of pro-inflammatory cytokines [86]. Indeed, in the lungs of rats the exposure to cigarette smoke stimulates transcription of inflammatory genes through a chromatin remodeling process [87]. However, in humans the most relevant cell types involved in COPD pathogenesis are macrophages and neutrophils, both secreting chemokines and matrix metalloproteases [88]. To elucidate the role of Sirt1 in macrophages infiltrating the lung of COPD patients, peripheral blood samples were collected and compared with those of non-smokers and smokers without COPD [89]. Sirt1 level and activity significantly decreased in smokers, and COPD patients and this evidence has been associated with the post-translational modification of Sirt1, including the introduction of 4-hydroxy-2-nominal (4-HNE) modification and the nitration of tyrosine. In parallel, a significant increase of the activity of NFκB subunit p65/RelA has been observed in macrophages and endothelial cells often associated with the release of significant amounts of IL-8 [89]. Whether Sirt1 plays a central role in this context remains to be determined. However, it must be noted that different results have been obtained studying cells coming from large and small airways, such as macrophages, lymphocytes and endothelial cells [90]. In a study, the following groups of patients were compared: COPD smoker, smokers without COPD and healthy non-smokers. It has been observed that in the large airways of COPD patients and smokers, Sirt1 expression was lower than in healthy people. On the contrary, in the small airways of COPD patients, a significant reduction of Sirt1 levels was found compared to the lung of smokers and healthy controls [90]. In addition, the exposure to chronic CS, in COPD patients, enhanced the expression of matrix metallo-proteinases 2, 9 and 12 and this phenomenon was associated with further reduction in Sirt1 level [91]. Interestingly, the activity of MMP9 is under control of the tissue inhibitors of MMP (TIMPs), which function is regulated at N-terminal lysine acetylation level and depends on Sirt1 [92]. Hence, as for other chronic non-transmissible diseases, in COPD Sirt1 and TIMPs could be considered as potential novel pharmacological targets [91]. 

Circulating endothelial progenitor cells (EPCs) have been studied in in the blood of COPD patients. Also here, emerged clearly the downregulation of genes, such as Sirt1 mRNAs, CD31, CD34, and miR-126-3p while the overexpression of miR-34a, a markers often associate with aging, was observed only in comparison with signals from healthy smokers [93]. Remarkably, Sirt1 protein and its fragments have been often found in human COPD sera [94]. Specifically, Sirt1 120KDa (S120) has been found fragmented in COPD patients compared with healthy smokers or non-smokers subjects [94]. Taken together, these observations suggest that CD31, CD34, Sirt1 mRNAs, miR-126-3p, miR-34a [93] and the S120fraction of Sirt1 [94] could be used as molecular biomarkers for a better diagnosis of COPD. 

In human bronchial epithelial cells, the expression of an antioxidant enzyme, the heme-oxygenase-1 (HO-1), is normally very low [95]. However, the exposure to CS increases HO-1 level through activation of the NFE2-related factor 2 (Nrf2) [95]. Remarkably, HO-1 level is controlled by Sirt1. Consistently, it has been shown that the neutrophil elastase does not block Nrf2, but it can cleave Sirt1 in lung epithelial cells resulting in a CS-induced downregulation of HO-1 [95]. 

Frequently, in COPD patients, cognitive impairment is observed. A study explored the use of Dl-3n-Butylphthalide (NBP), a plant extract known for its therapeutic effects on ischemic strokes, in a COPD cognitive impairment rat model [96]. As a result, the Sirt1/PGC-1α pathway was activated and slowed the progression of cognitive degeneration in animals [96]. In spite of its generally positive effect in COPD, it must be said that a Sirt1 polymorphism, detected in Turkey in the Muğla population, has been recently associated with COPD exacerbation. Specifically, the AG polymorphism in the rs7895833 locus and the CC in rs2273773 have been found more frequently recurrent in COPD patients than the normal GG AG and TT TC [97]. Hence, as anticipated for other conditions, also in COPD the role of Sirt1 remains controversial and additional studies are necessary to achieve a better understanding about the general involvement of Sirt1 in the disease pathogenesis and, more in general, about the value of Sirts as targets for the development of novel COPD therapeutic strategies. 

About other sirtuins, Sirt4 was down-regulated in human pulmonary microvascular endothelial cells treated with CS extract, meanwhile the expression of E-selectin, and that of the vascular cell adhesion molecule 1 (VCAM1) significantly increased [98]. Once activated, the attachment of monocytes to lung endothelium promotes the development of a pro-inflammatory environment [99]. In this context, the overexpression of Sirt4 seems sufficient to prevent the inflammatory reaction. Indeed, Sirt4 inhibits the degradation of IκBα, an inhibitor of NfκB [98] preventing the activation of the inflammatory cascade and acting along its pathway may represent a novel therapeutic approach to COPD progression. Whether Sirt4 could be considered as a promising target for COPD treatments needs to be ascertained (see Table 3).

## 6. Sirtuins in Neurodegenerative Disorders of Interest for Rehabilitation Medicine

The definition of neurodegenerative diseases encompasses a wide range of conditions, in which neuronal cells progressively lose their normal activity and die. This phenomenon alters mental function and often limits motility causing a progressive and incurable debilitation. These diseases are of interest for rehabilitation medicine although the molecular mechanisms underlying the positive effect that physical and cognitive training has on these conditions are still unresolved. The most important mental disorder is dementia, which affects a significant number of older adults over age of 85. Alzheimer’s disease represents 60–70% of all cases of dementia [100] whereas other relevant neurodegenerative pathologies are Parkinson’s disease, motor neuron diseases, prion disease, Huntington’s disease, spinocerebellar ataxia and spinal muscular atrophy, to mention only some of the most important neurodegenerative conditions. More in detail, Alzheimer’s disease (AD) is characterized by deposition of brain amyloid plaques and neurofibrillary tangles, which rise in consequence of amyloid precursor protein (APP), presenilin 1 (PS1), presenilin 2 (PS2), tau protein deposition and apolipoprotein E (APO E) gene mutations or altered processing. These proteins accumulate into the extracellular matrix forming deposits with consequences on matrix homeostasis and interfere with physiological impulse transmission [100]. In this disorder, it has been suggested that Sirt1 is probably the most important sirtuin involved in neuroprotection, which prevents or reduces accumulation of β amyloid. Here, neuronal Sirt1 overexpression or neuronal Sirt1 resveratrol-dependent activation decreased a Ser/Thr Rho kinase, which is involved in amyloid β metabolism [101]. Additional studies demonstrated that the deacetylating effect of Sirt1 on Tau’s lysine made this molecule accessible to ubiquitin ligases driving Tau to degradation significantly slowing the progression of the disease [102]. Further studies demonstrated the direct effect of Sirt1 on PI3K/Akt pathway determining Tau hyper-phosphorylation in a human neuroblastoma cell line. Here, Sirt1 downregulation by siRNA approach significantly decreased cell survival in consequence of inhibition of Akt phosphorylation. In this work, it was found that Sirt1 localizes close to the plasma membrane modulating Akt activity and decreasing β amyloid accumulation upon Tau hyper-phosphorylation [103].

Along an independent line of research, a recent study demonstrated the effect of Sirt3 on p53. In physiological condition deacetylated p53 prevents DNA damage and mitochondrial dysfunctions, however Alzheimer’s patients exhibit a decrease in Sirt3 associated with significant accumulation of acetylated p53 in the mitochondria of frontal cortex neurons. In experimental models, the direct deacetylation of mitochondrial p53 on lysine 320, exerted by Sirt3, seems to have neuroprotective effect on Alzheimer’s disease slowing its progression [104].

Interestingly new approaches to reduce AD symptoms may include physical and cognitive exercises. For instance, a recent clinical trial evaluated the feasibility of a music-supported video-based exercise in AD patients [105]. Exercises were progressively increased during a four weeks program, and physical parameters, such as warming up exercise, strength, static and dynamic balance, other functional exercises, endurance, and flexibility were recorded [105]. It was observed an amelioration in the exercise quality and at the end of the program patients were satisfied and enjoyed training [105]. Whether sirtuins activation, which normally occurs upon physical exercise, played a positive role in this context it remains to be ascertained. 

In developed countries, in the population over 60 years of age, it is common to find symptoms of Parkinson’s disease (PD). Typical signs of this pathology are tremors of head, hands and limbs, rigidity, bradykinesia and postural instability associated with cognitive and psychiatric disorders [106] without real therapeutic options. However, surgery and drugs can reduce symptoms and recently it was found that the application of transcranial alternating current stimuli significantly decreased motor symptoms [107]. As known, Parkinson’s disorder is determined by altered dopamine secretion caused by cell death in the *substantial nigra* and by the formation of Lewy’s bodies (intracellular α synuclein accumulation) in living neurons [108]. In this condition, Sirt1 seems acting as a neuroprotector through the activation of the heat shock factor 1 (HSF1), which enhances transcription of molecular chaperones, such as the heat shock protein 70 [109]. In PD, the effect of Res on Sirt1 and AMPK determines an increased mitophagy in dopaminergic neurons. In fact, Res stimulates the clearance of injured mitochondria and enhances degradation of α synuclein through autophagy thus inhibiting the formation of Lewy’s bodies [110]. In addition, in a PD mouse model, it was demonstrated the regulation of Sirt1 by Cdk5 through the ubiquitin-proteasome pathway. Here Cdk5 seems more expressed than in normal controls contributing to the loss of neuronal reactivity [111]. Interestingly, a small molecule called AGK2, which was identified as a potent Sirt2 inhibitor, showed a neuroprotective effect rescuing neurons from the toxicity consequent to α synuclein accumulation [112]. The same effect was obtained in vivo in a Sirt2 deficient mouse model [113]. In light of this contradictory results it remains unclear whether sirtuins play a positive or negative role in PD and further studies are necessary to elucidate this point.

Recent in vivo and in vitro studies indicated that microRNAs (miRs) regulate Sirt3. Specifically, miR-494-3p, which is enriched in PD neurons, binds Sirt3-3′UTR determining Sirt3 downregulation, which is associated with motor neuron impairment in a PD mouse model. The discovery of this Sirt3/miR-494-3p circuitry can be of interest to find a treatment for PD by using miR-494-3p as a target [114].

A large body of evidence suggest for a significant improvement of PD symptoms after physical activity. One of the most recent trials introduced “Ai Chi” exercises, a Japanese aquatic therapy, to the rehabilitation program for mild and moderate PD patients [115]. After five weeks of training, significant improvement in motility, balancing, and quality of life was observed compared to the group just following a land-based program [115]. This study suggests that this discipline, instead of the traditional exercises, might have a positive effect reducing PD symptoms. Whether molecular mechanisms leading to sirtuins activation are involved in this effect remains to be clarified. However, recent studies realized in the PD mouse model suggested for an involvement of Sirt1 as a neuro-protector agent activated by aerobics or some other physical exercises [116]. In this case, it was shown that the activity of Sirt1 and that of the mitochondrial complex I, both decreased in the hippocampus of PD mice, were rescued upon exercise improving the general physical conditions of the animals [116]. Moreover, in trained PD mice, lower levels of pro-inflammatory cytokines were observed suggesting for an action of Sirt1 on NF-κB pathway, which resulted inhibited [116]. Taken all together, these findings positively indicate that physical exercise might have an epigenetic effect increasing sirtuins activity in the presence of clear PD symptoms, leading to their amelioration.

Nowadays, the most common type of motor neuron disease is the amyotrophic lateral sclerosis (ALS). ALS is caused by a slow and progressive neurodegenerative process of neurons in the brain and in spinal cord resulting in loss of coordination, speech, eating and even breathing. The most common form of ALS is the sporadic one which affects about 90–95% of all ALS cases [117] although a less common familial form has been reported [118]. The pathogenesis of ALS is still not well understood, but it seems linked to a wide number of mutations detected in various genes, including superoxide dismutase (SOD) [119], TAR DNA-binding protein 43 [120], FUS RNA binding protein [121] and C9orf72 [122] to name few of the most commonly reported. To a better understanding of the role of sirtuins in ALS, some experiments were performed, in which Sirt1 was stimulated using Res. In these experiments, rat neuronal cells were incubated with ALS patient cerebrospinal fluid (CSF) or with CSF from a healthy donor. As a result, rat neuronal cells exposed to ALS fluid were more damaged than controls. However, whenever resveratrol had been added, the toxic effect of ALS-CSF was greatly reduced [123]. As in Parkinson’s disease, in ALS, it was demonstrated that Sirt1 deacetylates the heat shock factor 1 (HSF1) upregulating hsp70 and hsp25 prolonging the lifespan of motor neurons [124]. Another sirtuin, the mitochondrial Sirt3, was shown to be neuroprotective in ALS studies. In particular, mutation in SOD1 causes mitochondrial dysfunction and fragmentation often associated to ALS. A transgenic mouse model expressing a mutated SOD1 was used to test Sirt3 efficiency [125]. Remarkably, in motor neurons, Sirt3 overexpression protected against the dysfunctional consequences determined by a mutant SOD1 thus preventing mitochondrial damage and neuronal cell death [125].

A number of clinical trials showed the benefit of physical exercise in ALS patients. In a recent study, a significant increase of the score of the functional independence scale was observed in ALS patients after a specific program training, in which improvement of oxygen consumption, muscle strength and fatigue was reported [126]. Moreover, in an ALS-like condition observed in old people [127], physical exercise determined re-innervation of muscle fibers at neuromuscular junctions ameliorating symptoms [127]. Whether these beneficial effects might be consequence of sirtuin activation is still unclear. Similarly, as it will emerge clearly from what discussed below, also in Huntington’s disease (HD) the role of sirtuins is still one of the most controversial. HD is an autosomal dominant illness characterized by loss in coordination and motility, variable personality and psychiatric disturbances combined with cognitive dysfunction. The patients’ genotype is characterized by CAG repeats expansion that encode glutamine residues in the Huntingtin protein, which aggregates in the cytoplasm of neurons [128]. This illness is highly disabling, and first symptoms arise commonly in middle-aged adults (35–44 years) [129]. Some HD features are similar to other neurodegenerative diseases of old people, such as the accumulation of aggregates, cognitive decline and behavior changing [129]. A significant neuroprotection effect was obtained from Sirt1 overexpression in *C. elegans* after induction of polyglutamine cytotoxicity [130] However, in Drosophila, Sirt1 and Sirt2 downregulation was associated to neuron survival apparently suppressing the disease [131]. In contrast, in a transgenic mouse model overexpressing a truncated N-terminal Huntingtin protein, deletion of the Sirt1 catalytic site worsened Huntington’s disease symptoms while the overexpression of Sirt1 reduced protein aggregation improving physiological conditions [132]. Mechanistically, it has been proposed an effect of Sirt1 on TORC1 deacetylation, which normally interacts with CREB. In fact, in the Huntington mouse model, TORC1-CREB complex is interfered by mutated huntingtin protein and transcription of the brain-derived neurotrophic factor supporting neuronal growth, survival, and differentiation, cannot be transcribed [132]. The upregulation of Sirt1 rescues the formation of TORC1-CREB complex [132]. The role of Sirt2 was also investigated in the Huntington mouse model, but its downregulation did not have effects on the disorder and did not changed Huntingtin protein intracellular stacking [133]. However, Sirt3 activation upon stimuli with viniferin increased AMPK function and in consequence the neurodegeneration was attenuated [134].

In the attempt of finding a therapy that could ameliorate HD symptoms, the effect of β-Lapachone (βL), a naturally derived compound present in the roots of *Tabebuia avellanedae*, known to be beneficial as anti-bacterial, antiviral, anti-cancer, anti-inflammatory and beneficial for wound healing too, was verified in the HD mouse model [135]. The HD animals were injected with βL, and Sirt1, CREB, and PGC-1α expression, modification, and activity determined. In the brain of treated mice, it was found that Sirt1 and p-CREB expression and function increased, while PGC-1α acetylation was reduced. Altogether, treated animals showed an improvement of the rota-rod score compared to HD controls and reduction of Huntingtin protein deposit with consequent amelioration of the HD phenotype [136]. 

Recently, the introduction of a rehabilitation program has been suggested for HD patients. A clinical trial positively evaluated the effect of physical activity on speed, walking, balancing, muscular strength, and pulmonary and cognitive functions [137]. It is currently unknown whether the exercise program activated the sirtuin pathway. However, because HD is a genetic illness, the potential activation of Sirt1 could only provide palliative effects, waiting for the definitive correction of the genetic defect. See Table 4.

## 7. Conclusions

Epigenetics is a new field constantly under investigation, in which DNA structure is involved in gene regulation without altering the primary genomic sequence. During human development, DNA undergoes numerous structural modifications, such as cytosine methylation or chromatin remodeling by the addition of multiple histone modifications, among which the best characterized are certainly the acetylation/deacetylation and methylation/demethylation of lysine residues. Physiologically, all of these changes drive cells to differentiation or introduce modifications in their functional state. 

In a large number of pathophysiological conditions; however, the alteration of chromatin structure ends up with changes in the normal pattern of gene expression. To restore the physiological state, a body of evidence supports that a disease-specific physical activity program may help modifying our epigenetic landscape, improving cardiovascular and respiratory function, muscle strength, gastrointestinal absorption, and the efficiency of the nervous and immune systems. Recent studies assigned a role for sirtuins, the class III HDAC enzyme family, in a large number of diseases, in which they were found down- or up-regulated, but in general not properly working. Hence, at least in specific pathophysiological conditions, including cancer or neurodegenerative diseases, the role of sirtuins seems quite controversial. Remarkably, numerous observations report that sirtuins’ expression and activity increases upon intense exercise training, contributing to the amelioration of physical and cognitive parameters, such as walking, memory, and muscular strength. With this review, necessarily limited to those aspects of the role of sirtuins more easily amenable to chronic non-transmissible diseases of interest for rehabilitation medicine, we attempted to raise attention toward the regulation of these NAD^+^-dependent deacetylases combined with physical training. In conclusion, more attention should be paid to sirtuins that should be investigated in greater detail in the context of physical rehabilitation training programs of illnesses associated with aging.

## Figures and Tables

**Table 1:** Sirtuins regulation and function in selected cancers.

Sirtuin	Cancer Type	Expression	Effects	Cell Type	Ref.
1	Colorectal	Down	Tumor suppressor	Mouse colorectal cancer	[38]
	Colorectal	Up	Oncogene	Human colorectal cancer cell line	[39]
	Breast	Up	Oncogene	Human diploid fibroblast	[40]
	Prostate	Down	Tumor suppressor	Mouse prostate cancer	[42]
	Bladder	Down	Tumor suppressor	Mouse bladder cancer	[42]
	Glioblastoma	Down	Tumor suppressor	Mouse glioblastoma	[42]
	Ovarian BRCA1 mutated	Down	Tumor suppressor	Mouse ovarian cancer	[42]
3	Lymphocytic leukemia	Up	Oncogene	Tumoral B-cells	[44]
	Breast	Up	Oncogene	Human breast cancer	[44]
	Colorectal	Up	Oncogene	Human colon cancer epithelium	[44]
	Renal	Up	Oncogene	Human cancer renal	[44]
	Hepatocarcinoma	Up	Oncogene	Human hepatic carcinoma	[44]
7	Breast	Up	Oncogene	Human breast cancer	[41]

**Table 2:** Regulation and functional role of sirtuins in cardiovascular disease.

Sirtuin	CVD Type	Expression	Effects	Cell Type	Ref.
1	Hearth failure	Down	Production of ROS *, vessel inflammation and atherosclerosis.	PBMCs * & endothelial	[50]
	Pressure overload	Up	Mitochondrial dysfunction.	Endothelial	[50]
	Ischemia/reperfusion injury	Down	Heart damage.	Cardiomyocyte	[59,60]
	Hypertrophy	Up	Cellular growth.	Cardiomyocyte	[21]
2	Hypertrophy	Down	Increasing cellular growth & inhibiting apoptosis.	Cardiomyocyte	[68]
3	Hypertrophy	Up	Inhibiting apoptosis.	Cardiomyocyte	[72,73]
	Friedrich ataxia	Down	Increasing mitochondrial permeability. Altered TGFβ1 signaling.	Cardiomyocyte	[70,71]
6	Hearth failure	Down	Increasing of IGF1 signaling.	Cardiomyocyte	[77]
	Hypoxia	Up	Stimulation of ROS and induction of apoptosis.	Cardiomyocyte	[78]
7	Hypertrophy	Down	Altered DNA repair and cell growth.	Cardiomyocyte	[80]

* ROS = Reactive oxygen species; PBMCs = peripheral blood monocyte cells.

**Table 3:** Regulation and function of sirtuins in chronic obstructive pulmonary disease.

Sirtuin	Pulmonary Disease	Expression	Effects	Cell Type	Ref
1	COPD	Down	Increasing inflammation.	Macrophages & neutrophils	[89]
		Less Down	Increasing inflammation.	Large airway epithelium	[90]
		Significantly down	Induction of a severe inflammatory state.	Small airway epithelium	[90]
4	COPD	Down	Increasing of inflammation	Endothelium	[98]

**Table 4:** Regulation and function of sirtuins in neurodegenerative disease.

Sirtuin	Neurodegenerative Disease	Expression	Effects	Cell Type	Ref
1	Alzheimer	Down	Inhibiting amyloid catabolism and Tau degradation.	Human CNS * neuron	[101,102,103]
	Parkinson	Down	Inhibiting transcription of molecular chaperones and reducing α-synuclein clearance.	Human dopaminergic neuron	[109,110]
	Amyotrophic lateral sclerosis	Down	Neuronal damage.	Human motor neuron	[123]
	Huntington	Down	Reduction in neuronal differentiation, survival and growth.	Mouse and human neurons	[132,133]
		Up	Neuronal survival.	Drosophila neurons	[131]
		Down	Polyglutamine cytotoxicity	Worm neurons	[130]
2	Parkinson	Up	Accumulation of α-synuclein.	Human dopaminergic neuron	[112]
		Down	Accumulation of α-synuclein.	Mouse dopaminergic neuron	[113]
3	Parkinson	Down	Motor neuron impairment.	Human dopaminergic neuron	[114]
	Amyotrophic lateral sclerosis	Down	Mitochondrial damage & neuronal death	Human motor neuron	[125]
	Huntington	Down	Decrease of AMPK * activity and neuronal dysfunction.	Human neuron	[134]

* CNS = Central nervous system; AMPK =**** 5′ adenosine monophosphate-activated protein kinase 1.
